# 생태체계 이론을 적용한 기혼 폐경 여성의 주관적 건강상태에 대한 궤적: 잠재성장모형을 이용한 종단연구

**DOI:** 10.4069/kjwhn.2022.05.24

**Published:** 2022-06-29

**Authors:** Eun Jin Kim, Ju-Hee Nho

**Affiliations:** College of Nursing, Jeonbuk National University, Jeonju, Korea; 전북대학교 간호대학

**Keywords:** Health status, Longitudinal studies, Postmenopause, Women, 건강상태, 종단연구, 폐경, 여성

## Abstract

**Purpose:**

This study investigated the trajectory of subjective health status in married postmenopausal women and aimed to identify predictive factors affecting subjective health status.

**Methods:**

Data were obtained from women who participated in wave 4 (2012) of the Korean Longitudinal Survey of Women & Families Longitudinal Study and continued to the latest phase (wave 7, 2018). A latent growth model (LGM) was used to analyze data from 1,719 married postmenopausal women in the framework of the ecological system theory.

**Results:**

The mean age of the participants at wave 4 was 56.39±4.71 years, and the average subjective health status was around the midpoint (3.19±0.84). LGM analysis confirmed that subjective health status decreased over time (initial B=3.21, slope B=–0.03). The factors affecting initial subjective health were age, body mass index, frequency of vigorous physical activity (microsystem level), marital satisfaction (mesosystem level), and medical service utilization (macrosystem level). Medical service utilization and the frequency of vigorous physical activity were identified as predictive factors affecting the slope in subjective health status. The model fit was satisfactory (TLI=.92, CFI=.95, and RMSEA=.04).

**Conclusion:**

This analysis of the trajectory of subjective health status of married postmenopausal women over time confirmed that subjective health is influenced by overall ecological system factors, including the microsystem, mesosystem, exosystem, macrosystem, and chronosystem. Therefore, it is necessary to assess physical activity and support policies promoting access to medical services in order to improve the subjective health status of married postmenopausal women.

## Introduction

난소 기능의 상실로 인해 폐경 여성은 다양한 신체적, 정신적인 문제를 경험한다[1]. 폐경은 특히 여성이 가정에서 중심이 되어 가족 구성원들의 생계를 꾸리거나 책임을 지는 등의 중요한 역할을 하는 시기에 발생하므로, 여성의 삶에 있어 폐경에 대한 경험은 고통스러울 수 있다[2]. 폐경을 겪는 기혼 여성들은 포괄적이고 지속 가능한 지원을 배우자로부터 받고 있고[3], 가족의 틀 안에서 병리적인 문제가 나타나면 가족 구성원들의 건강에도 부정적인 영향을 준다[4]. 폐경 후 기간은 여성 삶의 절반 또는 3분의 1을 차지하고 있어[5], 이 시기의 건강상태 중요성이 강조된다.

주관적 건강상태란 건강의 다차원적인 측면을 반영하는 유용한 개념이며[6], 타당성과 신뢰성으로 인해 최근 많은 연구에서 더 많이 사용하는 지표가 되었고, 신체적 및 정신적 측면을 통합하여 실제 건강상태와 건강 목표를 강하게 연관시킨다[7]. 선행연구에 의하면 폐경 여성 중 41.6%가 자신의 건강상태를 부정적으로 평가하는 것으로 확인되었다[8]. 폐경 여성의 주관적 건강상태에 영향을 미치는 요인은 연령, 직업, 교육 정도, 배우자, 신체활동[8], 소득, 사회•경제적 지위(socioeconomic status)로[9] 확인된 바 있으나, 폐경에 영향을 미치는 요인은 사회•문화적 배경에 따라 다르고, 건강상태와 건강 행동 요인이 폐경의 강력한 예측 변수가 되므로[10] 폐경을 겪는 기혼 여성의 주관적 건강상태에 대한 체계적인 이해가 필요하다.

생태체계 이론(ecological system theory)은 개인과 환경은 상호작용을 통해 서로에게 영향을 미친다는 전제 하에 개인의 삶에 직∙간접적 영향을 미치는 환경과의 관계를 가까운 것에서부터 먼 것까지 모형으로 나타내는 이론으로, 미시체계(microsystem), 중간체계(mesosystem), 외체계(exosystem), 거시체계(macrosystem)및 시간체계(chronosystem) 변인으로 나누어진다[11]. 개인은 생태체계의 중앙에 위치하며, 미시체계, 중간체계, 외체계, 거시체계가 순서대로 개인을 둘러싸고 있다[11]. 미시체계는 개인을 둘러싼 가까운 주변을 의미하며, 연령, 건강[11]을 포함한다. 중간체계는 개인이 적극적으로 참여하는 환경을 의미하며 배우자와의 관계 만족[12]을 포함하고, 외체계는 개인의 직접적인 환경과 개인이 직접적이고 적극적인 역할을 하지 않는 사회적 환경 간의 연결을 의미하며, 이웃 및 커뮤니티 내에서 가족의 사회적 통합으로[13], 사회참여 활동을 포함한다[14]. 거시체계는 광범위한 사회•문화적 환경, 이념, 신념을 의미하며[11], 중간체계 및 외체계 등과 상호작용하여 각 체계의 요인을 구성하는 데 영향을 준다. 거시 시스템 수준의 중단, 부패 및 실패는 지원하는 개인, 가족, 그룹 및 커뮤니티의 문제를 악화시키며[15], 간호∙간병 통합서비스 이용[16]과 건강 관리 서비스 이용이 포함된다[15]. 시간체계는 전 생애에 걸쳐 일어나는 변화와 사회 역사적인 환경을 포함하며, 시간의 경과와 연속적으로 일어나는 사건들이 개인의 성장에 영향을 미침을 의미한다. 이러한 생태체계 이론은 간호 연구에서 포괄적인 건강행동 변화 개입을 체계적으로 개발하고 평가하기 위해 적용하고 있고[17], 개인 및 환경 등 여러 체계를 기반으로 건강상태 수준을 개념화하는 데 유용한 이론적 기틀이 될 수 있다[17].

주관적 건강을 주요 개념으로 하는 선행연구는 대부분 독립변수와 종속변수의 차이를 단면적으로 분석한 것으로[6], 기혼 폐경 여성의 주관적 건강상태 궤적을 종단적으로 확인한 연구는 찾기 어려운 실정이다. 기혼 폐경기와 그 이후의 시기는 개인 내 요소뿐 아니라 대인 간의 미시체계 및 가정에서의 중간체계, 외체계인 사회적인 요소의 측면에서 변화를 겪는 시기이고, 불안정성이 특징이므로 종단적 연구를 통해 주관적 건강상태와 이런 요소들 간 차이를 밝히는 연구가 필요하다. 개인 삶의 변화를 파악하고자 하는 조사는 종단면 조사(longitudinal survey)자료가 용이하기에[18], 본 연구에서는 여성가족패널조사 종단 자료를 활용하여 기혼 폐경 여성의 주관적 건강이 시간에 따라 어떻게 변화하는지 확인하고자 한다. Bronfenbrenner [11]의 생태체계 이론을 토대로 기혼 폐경 여성의 주관적 건강상태에 영향을 미치는 생태체계 변인을 총체적으로 분석하여 기혼 폐경 여성의 건강 증진을 위한 간호실무에 기여하고자 하며, 구체적 연구문제는 다음과 같다. (1) 기혼 폐경 여성의 주관적 건강상태 궤적(trajectory)은 어떠한가? (2) 기혼 폐경 여성의 주관적 건강상태 초기 수준에 영향을 미치는 요인은 무엇인가? (3) 기혼 폐경 여성의 주관적 건강상태 변화율에 영향을 미치는 요인은 무엇인가?

## Methods

Ethics statement: Obtaining informed consent was exempted by the Institutional Review Board of Jeonbuk National University (2022-02-015) because this was secondary analysis of anonymized data from the Korean Longitudinal Survey of Women & Families Longitudinal Study.

### 연구 설계

본 연구는 우리나라 기혼 폐경 여성의 주관적 건강상태의 궤적을 확인하고, 주관적 건강상태에 영향을 미치는 예측 요인을 파악하기 위해 2012–2018년 여성가족패널자료 조사를 종단적으로 분석한 이차자료연구이다.

### 조사 자료 및 대상

#### 조사 자료

본 연구는 기혼 폐경 여성의 생태학적 요인이 주관적 건강상태에 미치는 영향을 파악하기 위해, 한국여성정책연구원에서 수행한 여성가족패널자료(Korean Longitudinal Survey of Women & Families Longitudinal Study)에서 수집되었다(https://klowf.kwdi.re.kr/portal/mainPage.do). 해당 자료는 여성의 생애주기(life cycle)변화를 큰 축으로 삼아 설계하였으며, 2008년부터는 2년 주기로 조사하였다. 본 연구는 4차, 5차, 6차, 7차 년도(2012년–2018년)의 자료를 한국여성정책연구원 홈페이지에서 다운받아 활용하였다[18]. 본 자료를 이용한 목적은 종단 자료로 설계되어 주요 변수에 대한 변화를 확인하기 용이하고, 전국 단위의 여성을 대상으로 확률비례 계통추출법과 계통추출방법으로 추출한 대규모 자료로 표본의 대표성이 확보되어 연구 결과를 일반화하기 용이하기 때문이다[18].

#### 조사 대상

**Figure 1.**
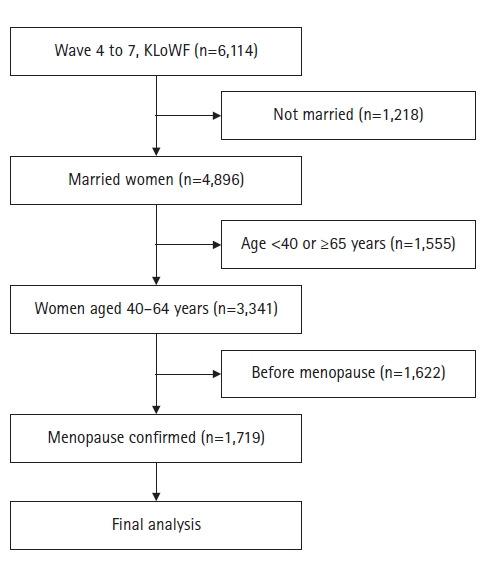
Flowchart of the study sample. KLoWF: Korean Longitudinal Survey of Women & Families Longitudinal Study.

여성가족패널조사의 조사 대상은 만 19–64세의 여성이며, 본 연구의 조사 대상은 여성가족패널 4차 년도 당시 기혼이며 폐경 상태인 여성으로 하였다. 폐경은 대륙, 인종, 유전학에 따라 다르며[9], 가족력, 흡연, 초경 연령, 임신 경험 여부, 구강피임약의 사용 여부, 사회•경제적 상태 등에 따라 차이가 있을 수 있지만 40–45세부터 나타날 수 있다[19]. 한국 여성의 자연적인 정상 폐경 연령을 보고한 선행연구[20]를 참고하여 본 연구에서 폐경 연령의 정의는 40세 이상인 응답자 중 본 연구의 폐경 대상자는 “폐경(마지막 월경이 있는지 1년이 지난 상태)을 경험하셨습니까”라는 질문에 ‘예’라고 응답한 대상자를 말한다. 여성가족패널 4–7차에 모두 응답한 대상자 6,114명 중 기혼여성 4,896명, 이 중 40–64세 여성 3,341명 중 폐경 상태인 1,719명을 대상자로 하였다(Figure 1).

### 측정 도구

#### 주관적 건강상태(subjective health status)

주관적 건강상태는 현재 본인의 주관적 판단에 따른 건강상태를 의미하며[18], 대상자 스스로 지각하는 건강상태를 측정하는 단일문항으로[21], ‘당신의 현재 건강상태는 어떻다고 생각하십니까?’라는 5점 리커트 척도로 구성된다. 본 연구에서는 역점수화하여(1점=매우 나쁘다, 2점=조금 나쁜 편이다, 3점=보통이다, 4점=대체로 좋은 편이다, 5점=매우 좋다), 점수가 높을수록 주관적 건강상태가 좋음을 의미한다.

#### 주관적 건강상태 궤적 예측 변수

선행연구를 통해 주관적 건강상태에 유의하게 영향을 미치는 변수로 확인된 연령[22], 체질량지수와 격렬한 신체활동 여부를 미시체계[8]로 구분하였고, 결혼만족감을 중간체계[10]로 구분하였다. 부부 동반 활동-사회봉사 빈도는 외체계[10]로 분류하였고, 의료서비스 이용 여부는 거시체계[23]로 구분하여 확인하였다. 2012년–2018년에 걸친 종단적 접근(chronosystem) [24]을 사용하여 기혼 폐경 여성의 주관적 건강상태 변화에 영향을 미치는지 파악하였다(Supplementary Figure 1).

**체질량지수:** 체질량지수는 본 조사에서 자가 보고된 키와 체중을 바탕으로 체질량지수를 산출하였고, 이를 아시아•태평양 기준에 따라[25] 저체중(<18.5 kg/m^2^), 정상(18.5–23 kg/m^2^ 미만), 과체중(23.0–25 kg/m^2^ 미만), 비만(≥25 kg/m^2^)으로 분류하였다.

**격렬한 신체활동 유무:** 격렬한 신체활동은 달리기, 등산, 빠른 속도로 자전거 타기, 빠른 수영, 축구, 농구 등의 체육 활동 및 무거운 물건 나르기 등의 활동을 의미한다. “최근 1주일 동안 평소보다 몸이 매우 힘들거나 숨이 가쁜 신체활동을 10분 이상 하신 적이 있으신가요? 있다면, 지난 1주일에 며칠 동안 하셨는지요?”의 질문에 ‘1점=1일, 2점=2일, 3점=3일, 4점=4일, 5점=5일, 6점=6일, 7점=7일(매일), 8점=전혀 하지 않았다’에 대한 응답이며, 본 연구에서는 ‘1점=한 적이 있다, 0점=한 적이 없다’로 재코딩하였다.

**결혼만족감:** 결혼만족감은 “요즘 결혼 생활에 대한 느낌을 가장 잘 표현하고 있는 숫자에 응답해 주십시오”라는 질문에 ‘매우 불행하다’ 1점에서부터 ‘매우 행복하다’ 10점까지의 응답을 말한다. 점수가 높을수록 결혼 생활에 대한 만족도가 높음을 의미한다.

**부부 동반 활동-사회봉사 참여:** 부부 동반 활동-사회봉사 참여는 “지난 한 달간 부부가 같이 사회봉사 및 공동체 참여하기를 얼마나 자주 하셨습니까?”라는 질문에 ‘1점=일주일에 2번 이상, 2점=일주일에 1번 정도, 3점=2주에 1번 정도, 4점=한 달에 1번, 5점=한 달에 1번도 하지 않았다’로 조사되었으며, 본 연구에서는 ‘0점=한 달에 1번도 하지 않았다, 1점=한 달에 1번, 2점=2주에 1번 정도, 3점=일주일에 1번 정도, 4점=일주일에 2번 이상’으로 역코딩하여, 점수가 높을수록 부부 사회봉사 활동 참여의 빈도가 높음을 의미한다.

**의료서비스 이용 여부:** 의료서비스 이용 여부는 “지난 1년 동안 건강상 문제로 의료서비스(병원 외래 방문, 입원, 건강검진 등)를 받아야 할 경우가 있었습니까?”라는 질문에 ‘1점=예, 0점=아니오’로 확인하였다.

### 자료 분석 방법

연구대상자의 인구학적 변수 및 주요 변수에 대한 기술통계를 실시하였다. 또한 2012년부터 2018년까지 기혼 폐경 여성의 주관적 건강상태 궤적에 영향을 미치는 요인을 살펴보기 위해 잠재성장모형(latent growth modeling) 분석을 실시하였다. 잠재성장모형은 반복 측정된 관찰치로부터 변화의 초기값(intercept)과 변화율(slope)을 추정하여 평균적인 변화궤적(trajectory)을 확인하고, 이러한 변화궤적 예측 변수를 밝히면서 변화 내 개인차를 설명할 수 있는 분석방법으로[26], 기혼 폐경 여성의 주관적 건강상태 궤적의 평균 초기값과 변화율을 확인한다. 잠재성장모형의 분석은 일반적으로 2단계를 거치며, 첫 단계는 비조건성장모형(unconditional growth model) 분석으로 독립변수를 배제하고 종속변수의 변화 추이를 추정하는 모형이며, 평균 변화 함수의 기울기와 절편을 추정한다. 그 다음 단계는 조건성장모형(conditional growth model) 분석이며, 1단계에서 얻은 잠재변수인 절편과 기울기를 다양한 설명 변수와 연결하여 종속변수의 개인차를 설명한다[27]. 본 연구에서의 종속변수인 주관적 건강상태에 영향을 주는 요인을 알아보기 위해 선행연구에서 유의한 영향을 나타낸 독립변수를 잠재성장모형에 통제변수로 설정하였다.

본 연구에서 사용한 주요 변수의 빈도 분석, 평균, 표준편차와 같은 기술 통계치는 IBM SPSS for Windows ver. 25.0 프로그램(IBM Corp., Armonk, NY, USA)을 이용하였고, 잠재성장모형 분석을 위해 AMOS ver. 23.0(IBM Corp.)을 사용했으며, 모형 적합도 평가를 위해 카이제곱(*χ*^2^) 통계량, 터커-루이스 적합지수(Tucker-Lewis index, TLI), 비교적합지수(comparative fit index, CFI), 근사오차 평균자승의 이중근(root mean square error of approximation, RMSEA) 지수를 사용하였고, 지수의 유의수준은 TLI와 CFI는 .90 이상, RMSEA는 .06 이하이면 모형이 적합한 것으로 판단하였다[28].

## Results

### 기혼 폐경 여성의 일반적 특성과 주관적 건강상태

여성가족패널조사의 4차, 5차, 6차, 7차 패널에 모두 응답한 1,719명의 4차 조사 시 인구사회학적 특성과 변수에 대한 기술통계 결과는 다음과 같다. 대상자의 평균 연령은 56.39±4.71세이고, 50대가 62.8%로 가장 많았으며, 체질량지수의 평균은 23.47±2.64 kg/m^2^로 정상 44.3%, 과체중 29.1% 순이었다. 격렬한 신체활동 빈도는 ‘예’가 24.0%, ‘아니오’가 76.0%였고, 결혼만족감의 평균은 6.41±1.62점이었다. 의료서비스 이용 여부는 ‘예’가 55.0%, ‘아니오’가 45.0%로 나타났다. 부부 동반 활동-사회봉사 참여는 ‘없음’이 94.0%, ‘한 달에 1번’이 3.4%, ‘일주일에 1번’이 1.5% 순이었다. 종속변수인 주관적 건강상태는 4차시 평균 점수는 3.19±0.84였고, 5차시에 3.23±0.85로 증가하였다가 6차시 3.14±0.82, 7차시 3.13±0.82로 점차 감소하는 경향을 보였다(Table 1). 주관적 건강상태의 왜도(range, –.40에서 –.33)와 첨도(range, –.41에서 –.24)는 모두 절대값 2 이내에 분포하여 정규성 분포를 만족하였다.

### 주관적 건강상태의 변화 궤적과 무조건성장모형 검증

**Figure 2.**
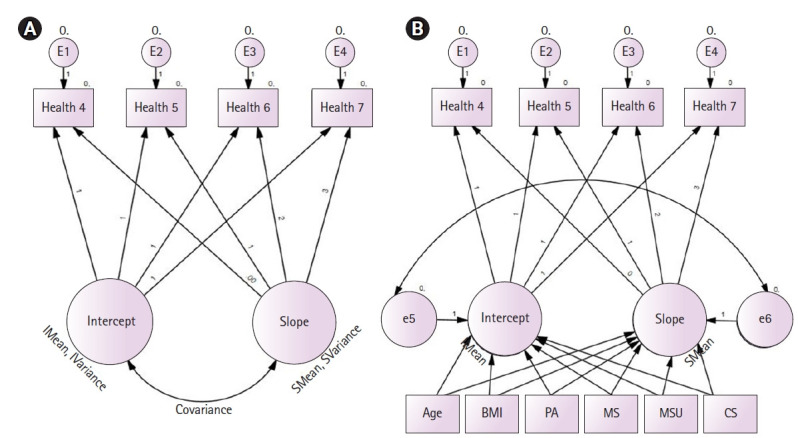
Growth models of this study. (A) Unconditional growth model. (B) Final conditional growth model. BMI: Body mass index; CS: community service; MS: marital satisfaction; MSU: medical service utilization; PA: physical activity.

기혼 폐경 여성의 주관적 건강상태 궤적은 선형 변화모형이 가장 적합한 것으로 나타났다. 이 모형의 적합도는 χ²=5.07 (df=5, *p*<.001), TLI .98, CFI .99, RMSEA .05로 모형이 적합하였다(Table 2). 이에 따라 선형 변화모형을 적용하였고, 기혼 폐경 여성의 주관적 건강상태 궤적에 대해 무조건모형분석(unconditional growth model)을 실시하였다. 본 연구에서 주관적 건강상태의 초기값(intercept)은 3.21±0.02 (*p*<.001)이며, 변화율(slope)은 –0.03 (*p*<.001)로 나타나 4차 년도(2012년)부터 7차 년도(2018년)에 이르기까지 주관적 건강상태의 수준은 점점 감소하고 있는 것으로 나타났으며, 한 차 년도 증가 시 –0.03만큼의 주관적 건강상태 수준이 감소하는 경향을 보였다. 또한, 주관적 건강상태의 초기값과 변화율 간의 공변량은 –0.03 (*p*<.001)로 부적(negative) 관계이며, 통계적으로 유의미한 것으로 확인되었다(Table 2, Figure 2).

### 조건성장모형을 통한 독립변수의 영향 요인 검증

본 연구의 생태학적 체계 독립변수가 기혼 폐경 여성의 주관적 건강상태 궤적에 어떻게 영향을 미치는지 분석하기 위해, 예측요인을 포함한 조건모형을 구축한 후 분석을 하였고, 모형은 χ²=4.01 (df=32, *p*<.001), TLI .92, CFI .95, RMSEA .04로 적합하였다(Table 3). 기혼 폐경 여성의 무조건모형분석을 통해 조건모형의 적용이 가능함을 확인했으며, 조건모형은 독립변수를 추가하여 독립변수의 영향을 검증하였다.

분석 결과, 기혼 폐경 여성의 주관적 건강상태의 초기값과 변화율에 유의하게 영향을 미치는 요인을 살펴보면 다음과 같다. 주관적 건강상태 궤적의 초기값에 영향을 미치는 요인으로 연령(B=-0.02, *p*<.001), 체질량지수(B=-0.02, *p*=.001), 격렬한 신체활동 여부(B=0.21, *p*<.001), 결혼만족감(B=0.06, *p*<.001), 의료서비스 이용 여부(B=-0.38, *p*<.001)가 통계적으로 유의미한 것으로 나타났다. 주관적 건강상태 변화율에 영향을 미치는 요인으로 격렬한 신체활동 여부(B=–0.06, *p*=.001)와 의료서비스 이용 여부(B=0.07, *p*<.001)가 통계적으로 유의미한 것으로 나타났다. 부부 동반 활동-사회봉사 참여는 주관적 건강상태의 초기값과 변화율에 통계적으로 유의미하지 않는 것으로 나타났다(Table 3, Figure 2).

## Discussion

본 연구에서는 기혼 폐경 여성의 주관적 건강상태 궤적 예측 변수를 확인하고 이에 영향을 미치는 미시체계 요인, 중간체계 요인, 외체계 요인, 거시체계 요인 및 시간체계 요인을 잠재성장모형에 투입하여 분석하였다. 그 결과는 다음과 같다.

첫째, 본 연구 대상자의 주관적 건강상태 궤적은 시간이 지남에 따라 감소하는 경향을 나타났다. 이는 시간이 지남에 따라 대상자의 나이가 증가하는 것과 관련이 있을 것으로 보인다. 기혼 폐경 여성의 초기 주관적 건강상태 수준이 높으면 연도별 선형 증가율이 낮게 예측되고, 초기 주관적 건강상태 수준이 낮을수록 연도별 선형 증가율이 높게 예측된다고 해석할 수 있다. 폐경 여성을 대상으로 한 선행연구에서 나이가 증가할수록 주관적 건강상태가 감소한다는 결과와 일치하는 결과이다[29]. 이와 같이, 점차적으로 주관적 건강상태를 나쁘게 인지하는 것은 건강 위험의 신호이며, 질병과 정신적 불건강으로 이어질 수 있기에[30], 반드시 세심한 관심과 배려가 필요하다.

둘째, 주관적 건강상태 초기 수준에 영향을 미치는 요인은 미시체계 요인 중 연령, 체질량지수, 격렬한 신체활동 여부이고, 중간체계 요인은 결혼만족감, 거시체계 요인으로 의료서비스 이용 여부로 확인되었다. 연령은 초기값만 부적(–)으로 주관적 건강상태 수준에 유의한 영향을 미치는 것으로 나타났다. 즉, 연령이 높아질수록 초기 주관적 건강상태 수준이 낮다는 것을 의미한다. 이는 폐경기 여성의 건강 수준은 폐경 전 건강 수준과 비교할 때, 낮은 건강 위험 수준에서 높은 건강 위험 수준으로의 전환을 염두에 두어야 한다는 연구 결과[31]를 뒷받침한다. 체질량지수 또한 초기값만 부적으로 주관적 건강상태 수준에 유의한 영향을 미치는 것으로 나타났다. 이는 연령이 낮을수록, 체질량지수가 낮을수록, 격렬한 신체활동을 할수록, 결혼만족감이 높을수록, 의료서비스 이용 여부를 안 할수록 주관적 건강상태의 초기 수준이 높은 것을 의미한다. 즉, 체질량지수가 높을수록 초기 주관적 건강상태 수준이 낮다는 것을 의미한다. 이는 여성의 체질량지수와 주관적 건강상태 사이에 유의한 역 연관성이 관찰되었다는 Hellgren 등[32]의 연구 결과를 지지한다. 격렬한 신체활동 여부는 주관적 건강상태의 초기값에 유의한 영향을 미쳤다. 즉, 격렬한 신체활동을 하는 사람이 하지 않는 사람보다 주관적 건강상태의 초기값이 높았다. 이는 폐경 여성 중 신체활동을 하는 사람이 신체활동을 하지 않는 사람보다 주관적 건강상태가 더 좋다는 연구 결과[33]를 지지한다. 중간체계 요인 중 결혼만족감은 주관적 건강상태의 초기값만 정적(+)으로 유의한 영향을 미치는 것으로 확인되었다. 이는 결혼만족감이 높으면 초기 주관적 건강상태 수준이 높아진다고 해석할 수 있다. 높은 결혼만족감은 더 나은 개인 건강과 건강행동과 관련이 있다는 Du Bois 등[34]의 연구와 맥락을 같이 한다. 생태학적 모델의 요소 중 폐경 여성의 건강 증진에 가장 도움을 많이 주는 요인은 개인에게 가장 밀접한 위치에 있는 대인관계 수준이므로[35], 건강상태를 향상하기 위해서 결혼만족감 같은 중간체계에서의 관계 수준을 높이는 전략을 고려해보아야 한다. 기혼 폐경 여성은 결혼만족감이 저하되면 주관적 건강상태 악화로 충분히 이어질 수 있으므로[4,36], 조직적 및 정책적으로 이를 개선하는 전략이 필요하다. 의료서비스 이용 여부는 주관적 건강상태의 초기값과 부적 관계로 통계적으로 유의미하였고, 이는 의료서비스 이용을 하는 사람의 초기 주관적 건강상태 수준이 유의하게 낮았음을 의미한다. 이는 자신의 건강상태를 나쁘다고 인식할 때 적극적인 건강 관리 활동을 탐닉하면서 건강 관리를 위해 의료서비스를 이용한다는 연구 주장[37]을 뒷받침한다.

셋째, 주관적 건강상태 변화율에 영향을 미치는 요인은 미시체계 요인 중 격렬한 신체활동 여부, 거시체계 요인으로 의료서비스 이용 여부로 확인되었다. 격렬한 신체활동을 할수록 주관적 건강상태 수준이 낮아지는 변화율이 나타났다. 이는 격렬한 신체활동을 할수록 주관적 건강상태의 변화율에 영향을 미쳐 주관적 건강상태의 수준이 낮아진다는 것을 의미한다. 격렬한 신체활동을 하는 대상자들의 초기 측정시점 평균 연령이 격렬한 신체활동을 하지 않는 대상자들보다 많았거나 초기 측정시점에서의 주관적 건강상태가 더 좋지 않았기 때문으로 생각된다. 격렬한 신체활동을 하는데도 주관적 건강상태의 변화율이 감소하는 것은 시간에 따라 연령이 많아질수록 자신의 주관적 건강상태를 낮게 인지하는 경우일 수도 있고, 만성질환, 사회적 활동[38]의 외생 변수가 동반되면서 주관적 건강상태 수준이 낮아졌을 수도 있다. 이를 통해 기혼 폐경 여성의 지속적인 신체활동을 통한 주관적 건강상태의 향상 전략 중 하나로 연령, 질환, 사회적 활동을 고려하는 차별적인 접근이 필요함을 알 수 있다. 또한, 폐경 여성들은 좌식행동 시간이 많아 신체활동량이 매우 부족한데[33], 이러한 부족한 신체활동량은 건강에 여러 문제가 되므로 조직적 또는 정책적 차원에서 이를 향상시켜야 할 것이다. 즉, 일상생활 속에서 신체활동을 유지하도록 문자 메세지나 모바일 동영상으로 동기와 자극을 주고[39], 부부들이 서로 가정에서 관심을 가지고 적극 지지해주거나, 신체적 건강상태를 개선하는 전략이 필요할 것으로 생각된다[40]. 본 연구에서의 신체활동량은 격렬한 신체활동을 1주일 동안 며칠 했는지에 대한 자가보고식 질문으로 측정되었다. 즉, ‘격렬한(vigorous)’이라는 단어에 대한 이해 여부를 파악하는 데 제한이 있고, 지난 1주일간 신체활동에 대한 대상자의 기억에 의존한 것으로 실제 대상자의 신체활동량과는 차이가 있을 수 있으므로[39], 추후 건강상태와 연관되는 객관적인 신체활동량을 측정한 추가 연구가 필요할 것이다. 의료서비스 이용 여부는 주관적 건강상태의 변화율에도 유의한 영향을 미침이 확인되었다. 의료서비스 이용 여부의 변화율은 양적 관계로 통계적으로 유의미하였는데, 시간이 지나면 의료서비스 이용을 할수록 주관적 건강상태 수준이 유의하게 증가하는 것을 의미한다. 즉, 의료서비스 이용을 할수록 주관적 건강상태의 변화율에 영향을 미쳐 주관적 건강상태의 변화 수준이 높아짐을 의미한다. 이는 폐경 진단 후 정기적으로 진료 및 검사 등의 의료서비스를 받는 것이 중요하다는 주장[40]을 뒷받침하였고, 시간이 지남에 따라 의료서비스를 이용하면 건강 관리가 잘 이루어지므로 대상자들의 주관적 건강상태 수준이 좋아졌을 것이라고 생각된다. 따라서, 의료서비스 이용 시 가족의 지지 전략과 국가 차원의 사회•정치적, 문화적, 환경적 프로그램과 체계적인 설정으로 개인의 직면한 건강문제에 긍정적인 변화를 제공할 수 있을 것이다.

연령은 대상자의 주관적 건강상태 수준의 변화율에서는 유의하지 않았는데, 이는 대다수가 여성 노인인 1인 가구의 주관적 건강상태의 변화율에 연령이 유의하지 않았다는 연구 결과[6]와 맥락을 같이 한다. 연령이 주관적 건강상태 수준의 변화율에 영향을 미치지 않았다는 것은 시간이 지남에 따라 다른 인구사회학적인 외생 변수가 작용하기 때문이라 여겨지며, 이를 확인하기 위해서는 추후 지속적으로 연령대별로 주관적 건강상태의 변화 궤적을 살펴볼 필요가 있겠다. 체질량지수도 주관적 건강상태 수준의 변화율에 유의한 영향을 미치지 않았다. 선행연구에 의하면, 폐경 여성을 대상으로 체지방과 건강상태와의 연관성이 유의하지 않다는 연구 결과[41]와 체질량지수가 높아질수록 건강상태가 악화한다는 상반된 연구 결과[42]가 확인되었다. 이처럼 상반되는 결과들이 있으므로 추후 체질량지수가 정상인 우리나라 폐경 여성들을 대상으로 한 연구를 고려해야 할 필요가 있겠다[37]. 또한, 폐경 여성은 부분적으로 피하지방에서 복강 내 내장지방으로 전환되는 대사 변화를 일으키기 쉽기 때문에[43], 대상자의 비만 정도를 체크할 떄 체질량지수뿐 아니라 허리둘레를 기준으로 한 복부 요소에 대한 고려도 필요할 것으로 보여진다.

외체계 요인 중 부부 동반 활동-사회봉사 활동 빈도는 주관적 건강상태의 초기값과 변화율 모두 통계적으로 유의하지 않은 것으로 나타났다. 그러나 다른 선행연구에서는 부부 동반 활동이 많아질수록 결혼만족도, 주관적 건강상태가 유의하게 좋아진다고 보고하였으므로[4,36], 추후 기혼 폐경 여성의 주관적 건강상태 향상을 위해 부부 동반 활동 프로그램을 적극 활용하는 전략이 필요할 것이다. 대중매체나 자원을 활용한 부부 동반 활동 전략을 적극 활용한다면 만족스러운 폐경 이후의 삶을 기대해볼 수 있을 것이다. 또한, 공식적인 사회집단에 참여하는 것이 더 높은 수준의 신체활동과 관련이 있으므로[44], 이러한 전략을 잘 활용하면 기혼 폐경 여성의 신체활동을 통한 주관적 건강상태의 지속적인 증가율을 높여줄 수 있을 것이라고 생각한다.

본 연구는 6년에 걸친 종단 연구의 데이터를 이용하였다. 표본의 대표성이 확보되었으며, 이 중 기혼 폐경 여성의 주관적 건강상태와 관련된 예측인자를 확인하였다. 따라서, 이들의 주관적 건강상태 궤적의 이해에 관한 기초를 형성하였다. 본 연구의 제한점은 자연 폐경과 조기 폐경을 구별할 수 없었다는 것이다. 또한, 선행연구에 근거하여 생태학적 모형에 따른 변수를 선정하였으나, 이차분석 연구로 제공된 자료에서 변수를 선정하게 되어 충분히 많은 변수들을 고려하지 못한 제한점이 있다. 그럼에도 불구하고, 본 연구는 생애주기에 있어 기혼 폐경 여성의 주관적 건강상태의 궤적을 생태학적 접근으로 살펴보았고, 구체적으로 미시체계, 중간체계, 외체계, 거시체계 및 시간적 체계로 주관적 건강상태의 예측 변수를 종단적 연구로 밝혔다는 데 연구의 의의가 있다.

본 연구 결과를 바탕으로, 기혼 폐경 여성의 주관적 건강상태의 궤적에 영향을 미치는 생태학적 요인들에 대한 지속적인 관심과 정책적인 전략이 필요하다는 결론에 도달할 수 있었다. 즉, 기혼 폐경 여성들의 건강상태 증진을 위해서는 기혼 폐경 여성뿐 아니라 배우자와 함께 부부 대상으로 간호 중재를 실행하고, 관련 기관의 정책적 요소를 결합한 생태학적인 접근으로 관리하는 노력이 필요하다. 기혼 폐경 여성의 주관적 건강상태를 예측할 수 있는 격렬한 신체활동에 대한 객관적인 측정과, 의료서비스를 이용할 때 검사 및 진료 예약 등 편리하게 이용할 수 있는 앱 개발이나 의료서비스 이용 체계의 편리성을 도모하는 정책을 제안한다.

## Figures and Tables

**Table 1.** General characteristics of participants at wave 4 (N=1,719)

Domain	Variable	Categories	Mean±SD or n (%)
Microsystem	Age (year)	Total	56.39±4.71
		40–49	130 (7.6)
		50–59	1,080 (62.8)
		60–64	509 (29.6)
	Body mass index	Total	23.47±2.64
		Underweight	37 (2.2)
		Normal	761 (44.3)
		Overweight	500 (29.1)
		Obese	421 (24.5)
	Vigorous physical activity	Yes	413 (24.0)
		No	1,306 (76.0)
Mesosystem	Marital satisfaction		6.41±1.62
Exosystem	Couple’s activities	Yes	103 (6.0)
	-Community service	Once a month	58 (3.4)
		Once every 2 weeks	14 (0.8)
		Once a week	25 (1.5)
		≥2 times a week	6 (0.3)
		No	1,616 (94.0)
Macrosystem	Medical service utilization	Yes	946 (55.0)
		No	773 (45.0)
Dependent variable	Subjective health status, Wave (year)	4 (2012)	3.19±0.84
		5 (2014)	3.23±0.85
		6 (2016)	3.14±0.82
		7 (2018)	3.13±0.82

**Table 2.** Means and variance of the initial intercept and the slope of the unconditional growth model (N=1,719)

Latent variable	B	SE	t	*p*	Model fit			
					χ² (*p*)	TLI	CFI	RMSEA
Initial intercept	3.21	0.02	172.72	<.001	5.07 (<.001)	.98	.99	.05
Slope	–0.03	0.01	–3.59	<.001				
Intercept-slope covariate	–0.03	0.01	–3.37	<.001				

CFI: Comparative fit index; RMSEA: Root mean square error of approximation; TLI: Tucker-Lewis index.

**Table 3.** Factors influencing the trajectory of subjective health status (N=1,719)

Variable	Intercept				Slope			
	B	SE	t	*p*	B	SE	t	*p*
Age	–0.02	0.01	–6.47	<.001	–0.01	0.01	–1.71	.087
Body mass index	–0.02	0.01	–3.24	.001	–0.01	0.01	–0.84	.402
Vigorous physical activity	0.21	0.04	5.25	<.001	–0.06	0.02	–3.36	<.001
Marital satisfaction	0.06	0.01	5.23	<.001	0.00	0.01	–0.02	.981
Couple’s activities-community service	0.03	0.04	0.94	.347	–0.01	0.02	–0.54	.590
Medical service utilization	–0.38	0.03	–10.99	<.001	0.07	0.02	4.63	<.001
Model fit: χ² (*p*)=4.01 (<.001), TLI=.92, CFI=.95, RMSEA=.04								

CFI: Comparative fit index; RMSEA: Root mean square error of approximation; TLI: Tucker-Lewis index.

## Supplementary materials

Further details on supplementary materials are presented online (available at https://doi.org/10.4069/kjwhn.2022.05.24).

Supplementary Figure 1. Conceptual framework of this study based on the ecological model. BMI: Body mass index.
