# Supplementary material for: Trajectories of subjective health status among married postmenopausal women based on the ecological system theory: a longitudinal analysis using a latent growth model
Source: Korean J Women Health Nurs. 2022 Jun 29;28(2):123–33. [Article in Korean] doi: 10.4069/kjwhn.2022.05.24 (PMC9334173; doi:10.4069/kjwhn.2022.05.24)
Supplement: Supplementary Figure 1. — Conceptual framework of this study based on the ecological model. BMI: Body mass index. [file kjwhn-2022-05-24suppl1.pdf]

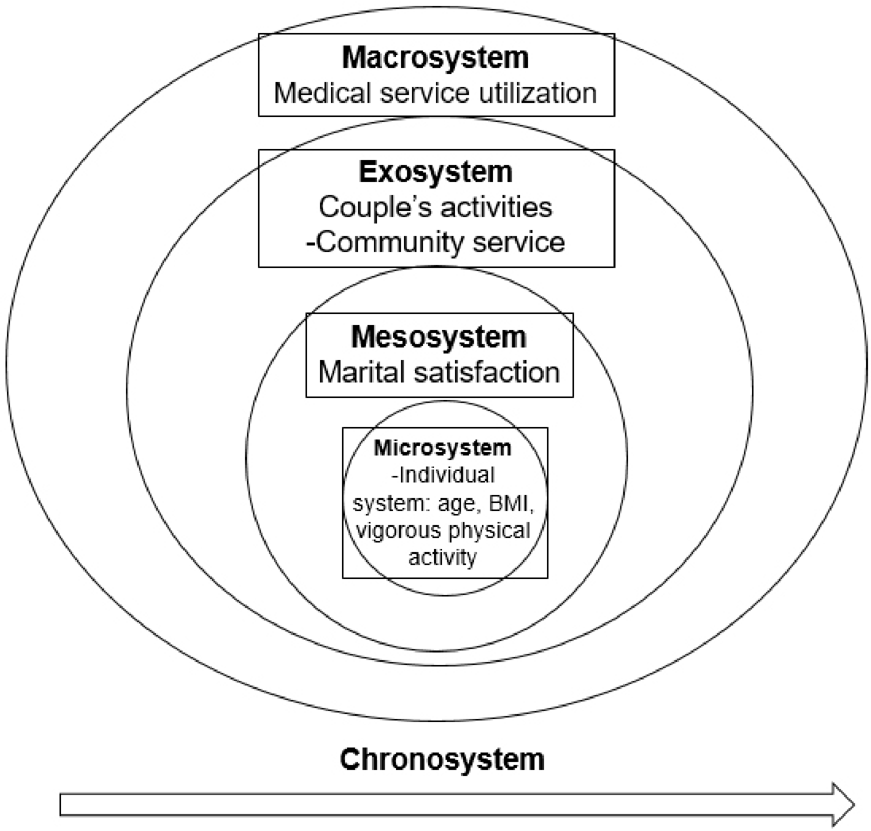

Supplementary Figure 1. Conceptual framework of this study based on the ecological model. BMI: Body mass index.
